# Controlling Amyloid Fibril Properties Via Ionic Liquids: The Representative Case of Ethylammonium Nitrate and Tetramethylguanidinium Acetate on the Amyloidogenesis of Lysozyme

**DOI:** 10.1021/acs.jpclett.2c01505

**Published:** 2022-07-28

**Authors:** Visakh V. S. Pillai, Pallavi Kumari, Srikanth Kolagatla, Victoria Garcia Sakai, Svemir Rudić, Brian J. Rodriguez, Marina Rubini, Katarzyna M. Tych, Antonio Benedetto

**Affiliations:** † School of Physics, University College Dublin, Dublin D04 N2E5, Ireland; ‡ Conway Institute of Biomolecular and Biomedical Research, University College Dublin, Dublin D04 N2E5, Ireland; § ISIS Neutron and Muon Source, Rutherford Appleton Laboratory, Science & Technology Facilities Council, Didcot OX11 0QX, U.K.; ∥ School of Chemistry, University College Dublin, Dublin D04 N2E5, Ireland; ⊥ Groningen Biomolecular Sciences and Biotechnology Institute, University of Groningen, 9747 AG Groningen, The Netherlands; # Department of Science, University of Roma Tre, 00146 Rome, Italy; ○ Laboratory for Neutron Scattering, Paul Scherrer Institute, 5232 Villigen, Switzerland

## Abstract

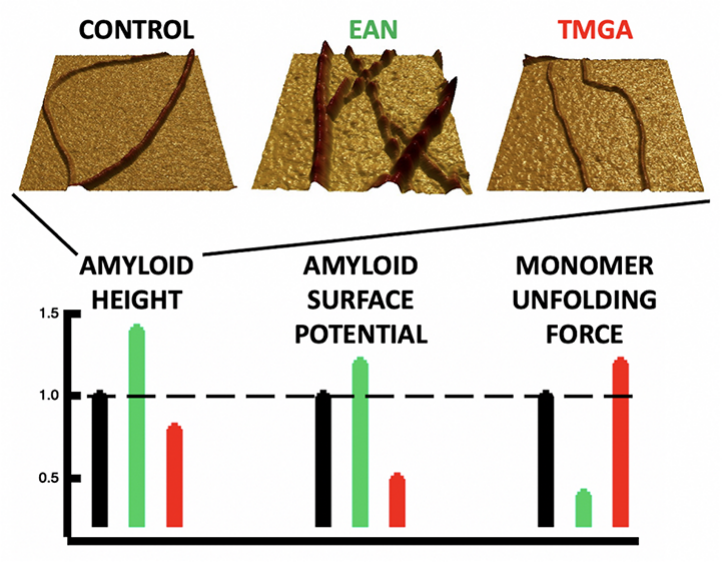

Protein aggregation into amyloid fibrils has been observed in several pathological conditions and exploited in nanotechnology. It is also key in several biochemical processes. In this work, we show that ionic liquids (ILs), a vast class of organic electrolytes, can finely tune amyloid properties, opening a new landscape in basic science and applications. The representative case of ethylammonium nitrate (EAN) and tetramethyl-guanidinium acetate (TMGA) ILs on lysozyme is considered. First, atomic force microscopy has shown that the addition of EAN and TMGA leads to thicker and thinner amyloid fibrils of greater and lower electric potential, respectively, with diameters finely tunable by IL concentration. Optical tweezers and neutron scattering have shed light on their mechanism of action. TMGA interacts with the protein hydration layer only, making the relaxation dynamics of these water molecules faster. EAN interacts directly with the protein instead, making it mechanically unstable and slowing down its relaxation dynamics.

Proteins are the molecular machines of life.^1^ They carry out a hugely complex variety of biochemical functions in living cells in addition to having a huge range of technological applications, including several antibacterial formulations^2^ and the new protein-based Covid-19 vaccines.^3^ Under specific physicochemical conditions, the majority of proteins undergo structural transformations leading to the formation of aggregated structures known as amyloid fibrils.^4−8^ Amyloid fibrils and, more generally, protein aggregation processes are involved in key biological mechanisms in healthy organisms^7−10^ and have also been observed in several diseases, including Alzheimer’s and Parkinson’s diseases.^7,8,11−13^ Moreover, amyloid fibrils have been exploited as advanced materials in biomedicine, tissue engineering, renewable energy, environmental science, nanotechnology and material science.^14−17^ The process that, starting from the functional folded protein monomers, leads to the formation of the mature amyloid fibrils is known as amyloidogenesis. For a given protein, different amyloidogenic pathways, characterized by different intermediate structures, such as oligomers and proto-fibrils, can be observed and lead to mature amyloid fibrils of different morphology and cyto-toxicity.^18−21^ For example, several studies have linked the cyto-toxicity of amyloid fibrils to the formation of specific oligomeric intermediates, transiently formed during the fibril assembly.^22,23^ As a result, being able to control amyloidogenesis can have important implications in health, since inhibiting the formation of the toxic intermediates can be exploited in effective therapeutics,^24^ and in material sciences, since tuning the morphology and elasticity of the amyloid fibrils can be exploited in advanced biomaterials.^17,25^

The amyloidogenic pathway is determined by the fine balance of several different interactions, including electrostatic and dispersion forces between protein residues, as well as entropic contributions to the total free energy coming from the protein solvation shell.^26^ For example, the addition of different concentrations of inorganic salts such as NaCl has shown to lead to different mature amyloid fibrils.^27^ In this context, ionic liquids (ILs), a relatively new and vast family of complex organic electrolytes, can play a decisive role. ILs consist of an organic cation and either an organic or inorganic anion^28^ and display a marked affinity toward biomolecules and biosystems,^29−31^ which has been already exploited in several applications, including pharmacology and drug delivery.^32−35^ Because of their extreme variety and tunability, also explored in several single protein-IL studies,^36^ ILs can actually offer a novel and vast landscape to control amyloidogenesis, potentially leading to new strategies against amyloid-based diseases and new opportunities in material sciences and nanotechnology. In the last 10 years, the effect of ILs on amyloidogenesis and mature amyloid fibrils has been the subject of several studies.^37−45^ The most important observation from these studies relevant to the work presented here is the observation that ILs can either inhibit or favor the amyloidogenesis. For example, it has been reported that ethylammonium nitrate (EAN) enhances the amyloidogenesis of lysozyme,^46^ while tetramethyl guanidinium acetate (TMGA) inhibits it (SI Figure 1).^47^ However, the microscopic mechanism behind these two opposite effects is still unclear. Its understanding would be the initial step toward the use of ILs to control protein amyloidogenesis and is the focus of this study. Herein, we present a comprehensive atomic force microscopy (AFM), optical tweezers, and neutron scattering investigation into the effect of EAN and TMGA on the amyloidogenesis of lysozyme.

First, the morphology of IL-incubated amyloid fibrils was studied with AFM. Specifically, the height distributions of the amyloid fibrils obtained by incubating the functional folded protein monomers, at 65 °C and pH 2.0 for 8 days in water solutions of the two ILs, have been compared with the amyloid fibrils obtained upon incubation in sole water. To assess the role of electrostatic versus dispersion interactions, the height distributions of the amyloid fibrils obtained upon incubation in NaCl–water solutions, prepared at the same ionic strength of the IL–water solutions, have been also measured and used as a benchmark. Different from the earlier studies, here the focus was on lower concentrations of ILs, ranging from one to five ILs per protein. For more details on sample preparation and methodology, please refer to the Supporting Information. Figure 1 shows representative AFM images of the lysozyme amyloid fibrils incubated in water and water solutions of EAN and TMGA at a molar ratio of 3.5 ILs per protein. Already by visual inspection, it was clear that the amyloid fibrils incubated in EAN and TMGA water solutions have different morphologies and are, respectively, thicker and thinner than the amyloid fibrils incubated in water. The average heights extracted from the height distributions of Figure 1 confirmed this picture. They were found to be 1.94 ± 0.05 nm in EAN solution, 1.12 ± 0.09 nm in TMGA solution, 1.30 ± 0.08 nm in NaCl solution, and 1.41 ± 0.11 nm in water alone. Additional measurements carried out on amyloid fibrils incubated for shorter times (i.e., for about 3 and 6 days) confirmed that after 8 days of incubation the mature amyloid fibril stage was reached for all the measured systems (SI Figure 2).

**Figure 1:**
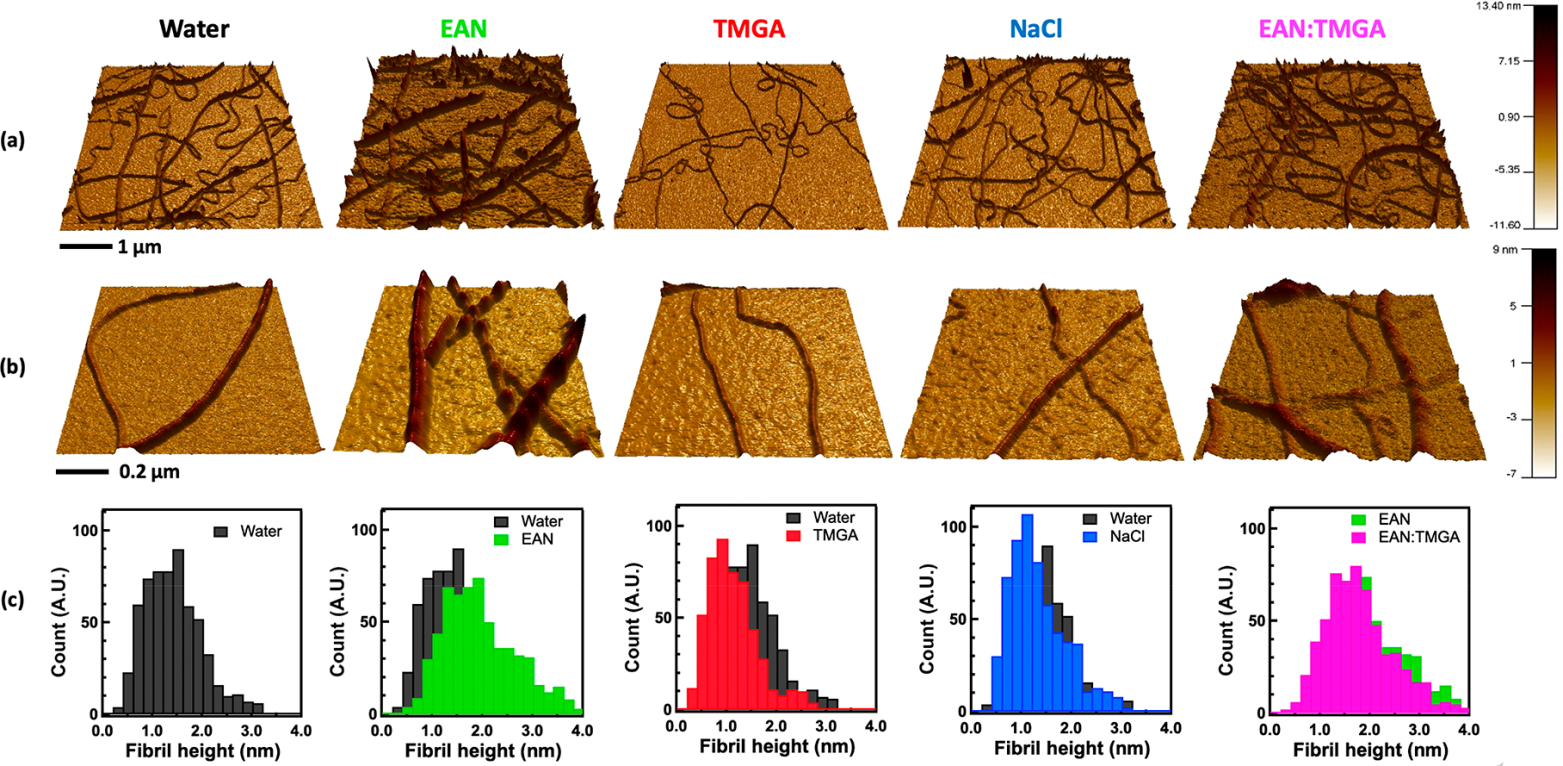
Representative height 3D AFM images (a), zoom images (b), and histogram distributions (c) of lysozyme amyloid fibrils incubated in sole water (black) and water solutions of EAN (green), TMGA (red), and NaCl (blue) at a molar ratio of 3.5 ILs per protein. The last column reports the results upon incubation in a water solution of a mix of the two ILs at a relative lysozyme:EAN:TMGA molar concentration of 1:1:3.5 (pink). The distributions in panel (c) are not limited to the data in panel (a) but have been obtained using all sets of AFM data (see the Supporting Information for more details).

The effect of IL concentration on the average height of the amyloid fibrils is reported in Figure 2 along with the NaCl case for comparison. By looking at the figure, the two opposite effects of EAN and TMGA become immediately clear: the presence of EAN leads to thicker amyloid fibrils, whereas the presence of TMGA leads to thinner ones. Interestingly, these two opposite effects already occur at the lowest measured concentration of one IL per protein. An additional set of AFM experiments in which the two ILs have been added to sole water-incubated mature amyloid fibrils did not show any effect on the amyloid fibrils’ height (SI Figure 3). Taken together, these results suggest that the driving mechanism occurs at the single-molecule level.

**Figure 2:**
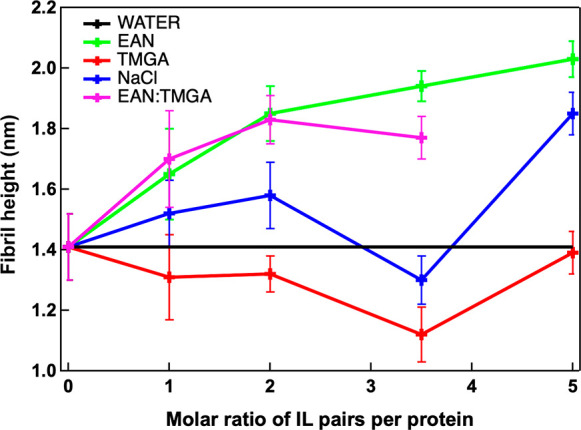
Average height values as a function of IL:lysozyme molar ratio for lysozyme amyloid fibrils incubated in water solutions of EAN (green), TMGA (red), and NaCl (blue) along with its value upon incubation in sole water (black line). The average height values obtained upon incubation in water solutions of a mix of the two ILs at relative lysozyme:EAN:TMGA molar concentrations of 1:1:*x* with *x* = 1, 2, and 3.5 are also reported (pink). Error bars are one standard deviation. Solid lines are only guides for the eye.

Different morphologies of amyloid fibrils are usually associated with different amyloidogenic pathways.^48−50^ In this context, thicker lysozyme amyloid fibrils are linked to an oligomer-based aggregation pathway, whereas thinner fibrils are linked to the earlier formation of proto-fibrils.^50^ On this basis, the AFM morphological results can be interpreted as suggesting the presence of two different amyloidogenic pathways for lysozyme in EAN and TMGA. The thinning effect of TMGA exhibits a minimum at a molar ratio of 3.5 ILs per protein, after which the amyloid fibril height starts to increase (Figure 2). A similar trend (i.e., presence of a minimum) was observed upon incubation in NaCl, suggesting that the lysozyme–TMGA mechanism of interaction could be strongly driven by its ionic character and by its interaction with the protein solvation shell. A completely different trend was observed in the EAN solution, suggesting that a direct interaction between lysozyme and EAN could be dominant in this case.

To compare these two different interaction mechanisms, amyloid fibrils obtained by incubating the folded functional lysozyme monomers in water solutions of a mix of the two ILs have been investigated. The experiments were performed at a lysozyme:EAN molar ratio of 1:1 and at lysozyme:TMGA molar ratios from 1:1 to 1:3.5. The results are shown in Figures 1 and 2. Already by visual inspection of Figure 1 it is clear that the morphology of the amyloid fibrils obtained upon incubation in the water solution of the two ILs when mixed is very similar to the thicker morphology of the EAN case. This suggests that, in the case of this mixture of ILs, it is the lysozyme–EAN mechanism of interaction that dominates the process of amyloidogenesis. This observation was confirmed by the IL-concentration dependence of the amyloid fibril height reported in Figure 2: for all the investigated concentrations, the mean height of the amyloid fibrils in the mixed IL–water solutions overlapped with the height of the amyloid fibrils in the EAN-only water solutions case. The predominant character of EAN over TMGA agreed well with the suggested picture for which EAN interacts with the lysozyme monomer directly, while TMGA affects mainly its solvation shell.

Different amyloid fibril morphologies and amyloidogenic pathways are usually associated with different properties, such as mechanical properties and surface charge distributions, and therefore lead to different degrees of interaction with other biomolecules and cells.^51,52^ In this context, the surface electric potential of the amyloid fibrils plays a key role in governing the amyloidogenic pathway and the interaction of the amyloid fibrils with other biomolecules and cells. It has been shown, for example, that the amyloid fibril height positively correlates with the surface electric potential of the amyloid fibril.^53^Figure 3 reports representative open-loop Kelvin probe force microscopy (OL-KPFM) images and the associated distributions showing the surface electric potential of the amyloid fibrils shown in Figure 1. The mean surface electric potentials extracted from these distributions were 1.67 ± 0.01 V in EAN solution, 0.67 ± 0.1 V in TMGA solution, 0.73 ± 0.05 V in NaCl solution, 1.57 ± 0.01 V in EAN:TMGA 1:3.5 mix solution, and 1.48 ± 0.01 V in sole water. These surface electric potential values have also been used to compute the “work function” of the samples, which corresponds to the minimum thermodynamic work needed to remove an electron from the sample surface (SI Table 1). The surface electric potential values correlate well with the average heights of the amyloid fibrils, confirming the presence of two different amyloidogenic pathways in EAN and TMGA solutions and potentially hinting at two different degrees of interaction of the resulting mature amyloid fibrils with biosystems and cells. Moreover, in the case of TMGA solution, the surface electric potential distribution shows the presence of an additional minor peak: a bimodal fit provided 0.64 ± 0.01 V for the main peak and 0.83 ± 0.01 V for the additional peak. The presence of this additional peak could be a signature of the presence of an additional small population of amyloid fibrils in the TMGA solution.

**Figure 3:**
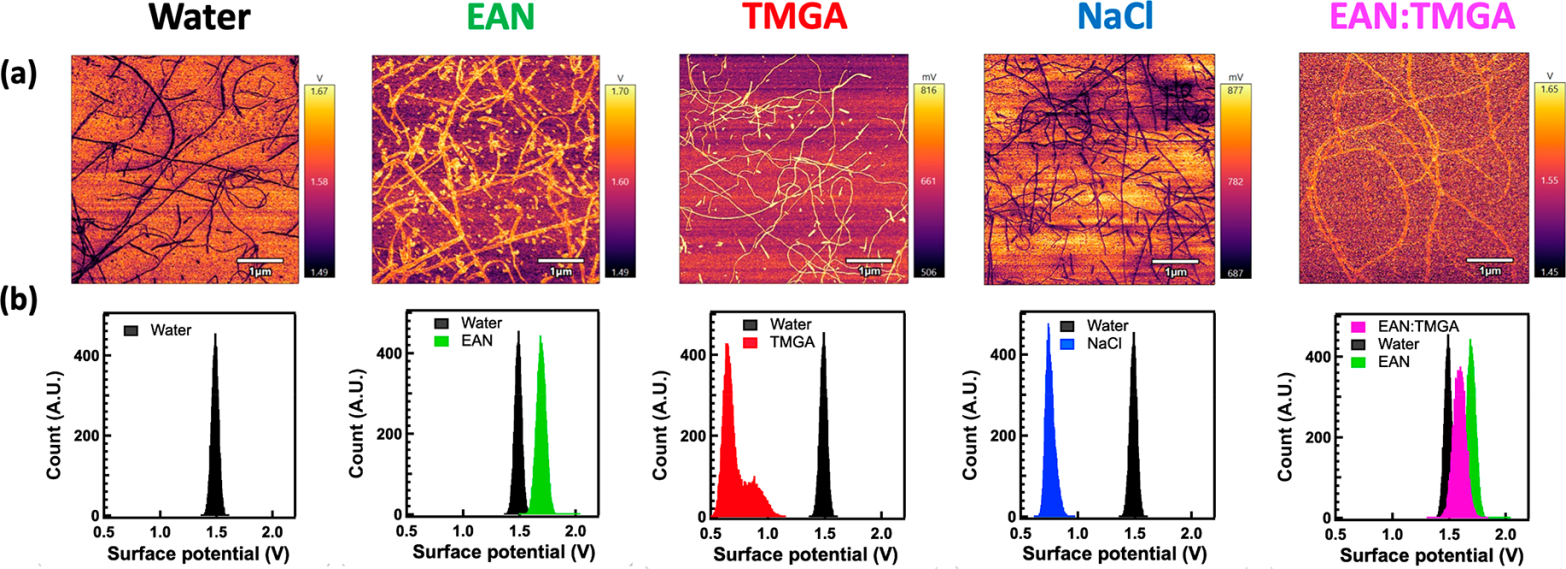
Representative surface electric potential OL-KPFM images (a) along with their associated distributions (b) for lysozyme amyloid fibrils incubated in sole water (black), water solutions of EAN (green), TMGA (red), and NaCl (blue) at a molar ratio of 3.5 ILs per protein, and in water solutions of EAN:TMGA at 1:3.5 molar ratio. The distributions in panel (b) are not limited to the data in panel (a) but have been obtained using all the sets of OL-KPFM data (see the Supporting Information for more details).

Because no additional peaks have been observed in the height distribution in the TMGA solution (Figure 1), we can conclude that this additional small population of amyloid fibrils differs from the main population only in its hydration/solvation layer. These two equilibrium surface potential configurations in TMGA suggest that TMGA would predominantly interact with the protein hydration layer.

To look at the single protein–IL interaction, hypothesized to be at the origin of the two different effects of EAN and TMGA observed at the amyloid fibril level, optical tweezers and neutron scattering were employed.

The force required to unfold the natively folded lysozyme monomers were measured with the optical tweezers in phosphate-buffered saline (PBS) buffer solutions of the two ILs and NaCl, at 5% salt concentration in all cases, and in pure buffer. For this investigation, a lysozyme mutant with only two cysteine residues, located at the N- and C-termini of the protein sequence, was expressed and functionalized for the optical tweezers measurements. Optical tweezers unfolding experiments were performed at a constant velocity of 500 nm/s. Please refer to the Supporting Information for more details on sample preparation and methodology. The average unfolding forces were found to be 15 ± 7 pN in EAN, 42 ± 7 pN in TMGA, 41 ± 7 pN in NaCl, and 37 ± 7 pN in pure buffer (Figure 4). As a result, the forces required to unfold the lysozyme monomers in the presence of EAN are significantly lower than in buffer alone, and in the presence of TMGA slightly higher, within error, than in buffer alone. Moreover, the unfolding forces in TMGA and NaCl were very similar. This trend correlated very well with the trend observed for the amyloid fibril height and surface electric potential and confirmed that the two ILs have two opposite effects also at the single-protein level, as hypothesized. Because protein unfolding is one of the initial steps of amyloidogenesis, the AFM and optical tweezers results suggest the following mechanism of action of the two ILs. EAN mechanically destabilizes the lysozyme monomers, making them easier to unfold favoring, perhaps, an oligomeric-based amyloidogenic pathway and leading to thicker amyloid fibrils. TMGA, instead, makes the lysozyme monomers slightly harder to unfold and leads to thinner amyloid fibrils, suggesting that it promotes an oligomeric-free proto-fibril amyloidogenic pathway. In this context, a mechanism similar to the one suggested here for TMGA has been recently observed for the prion protein.^54^ Moreover, our hypothesis that EAN interacts with the protein directly, whereas TMGA reacts with its solvation shell, was well supported by the relative variation of the unfolding force that is substantial in EAN (−60%) and quite modest (and within the error bars) in TMGA (+13%). To validate this interaction picture, neutron scattering experiments were performed.

**Figure 4:**
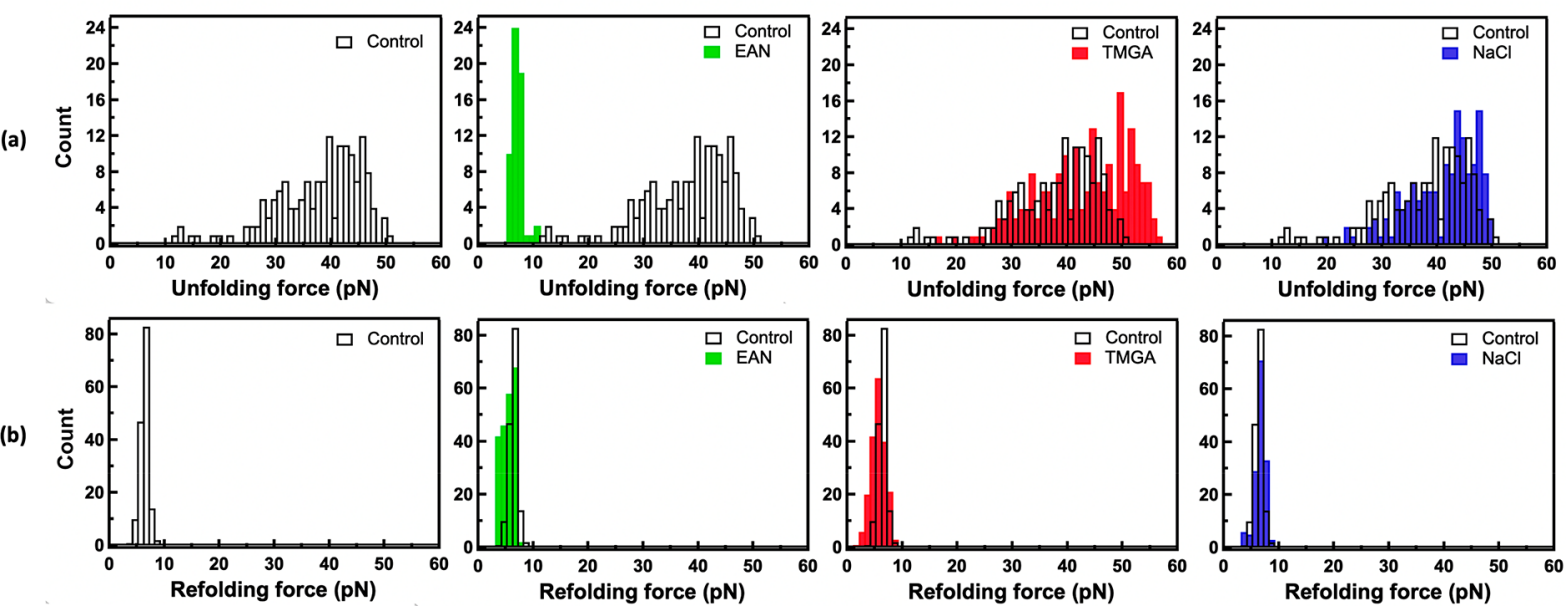
Average unfolding (a) and refolding (b) forces measured using single-molecule optical tweezers at constant velocities of 500 nm/s for lysozyme monomers in pure PBS buffer (black and white) and buffer solutions of EAN (green), TMGA (red), and NaCl (blue).

The elastic neutron scattering (ENS) profiles of lysozyme hydrated in water solutions of the two ILs and in water alone were collected as a function of temperature.^55^ The IL concentration was set at a molar ratio of 2 ILs per protein, in line with the AFM study. ENS can be considered as a highly precise molecular calorimeter. Upon increasing the temperature, any reduction of the ENS intensity indicates either an activation/enhancement of dynamical relaxations or the activation/enhancement of vibrational modes in the system. Moreover, the neutron scattering length density, i.e., the probability of a neutron scattering event, is very high for hydrogen atoms and differs substantially between hydrogen and deuterium atoms, allowing the contribution of selected hydrogen atoms to be masked via hydrogen-to-deuterium exchange. The ability to focus on selected hydrogen atoms by exchanging the other hydrogen atoms with deuterium atoms is quite unique to neutron scattering and was exploited here.^56,57^ For instance, two sets of samples were prepared: (i) one in D_2_O, in which the protein predominantly (>95%) contributes to the measured (incoherent) ENS intensity, and (ii) one in H_2_O, in which the protein hydration water also contributes (about 40%) to the measured ENS intensity. In both cases, the direct contribution of the ILs is negligible (<5%). Please refer to the Supporting Information for more details on sample preparation and methodology. In the set of samples in D_2_O (Figure 5a), the ENS profile of lysozyme in TMGA perfectly overlapped with the one in water alone, confirming that there is no direct interaction between TMGA and the protein. On the other hand, the ENS profile of lysozyme in EAN was shifted to higher intensities with respect to the one in water alone, confirming that EAN interacts directly with the protein. In the set of samples in H_2_O (Figure 5b), the ENS profile of the lysozyme–water in TMGA was shifted to lower intensities with respect to the one in water, confirming that TMGA interacts directly and solely with the protein hydration shell. The strong degree of interaction between TMGA and water was confirmed by inelastic neutron spectroscopy carried out using the water solutions of the two ILs (Figure 5c).^58^ From these measurements it emerged that TMGA has a significantly greater effect on water’s inelastic profile than EAN. More specifically, both the translational and librational bands of water are significantly affected by TMGA that also perturbs the bending and stretching OH bands of water.

**Figure 5:**
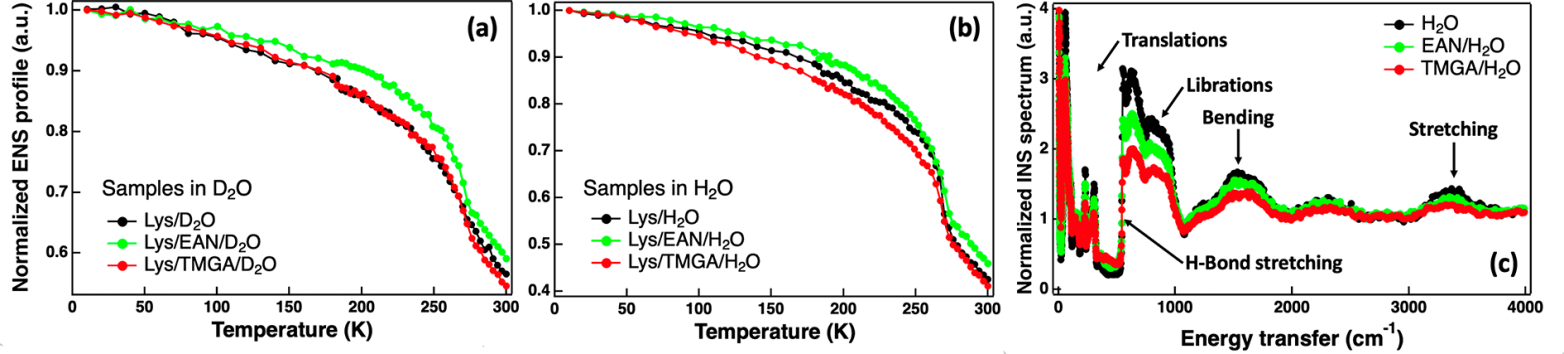
(a) Elastic neutron scattering intensity as a function of temperature for lysozyme hydrated in heavy water (black) and heavy water solutions of EAN (green) and TMGA (red) at a molar ratio of 2 ILs per protein. (b) As in panel a but with water instead of heavy water. (c) Inelastic neutron scattering spectrum of pure water (black) and water solutions of EAN (green) and TMGA (red) at 0.5 M.

In conclusion, we showed that EAN and TMGA offer a novel way to control the amyloidogenesis of lysozyme and tune the morphological and electrical properties of the mature amyloid fibrils (Table 1). The AFM study has highlighted the presence of two distinct amyloid fibril morphologies for EAN and TMGA, suggesting the presence of two different amyloidogenic pathways, both driven by single protein–IL interactions in which EAN interacted strongly with the lysozyme monomer while TMGA affected mainly the lysozyme hydration shell. Optical tweezers and neutron scattering investigations have supported this picture and allow formulation of the following explicative hypothesis for the two different effects observed with the two ILs: EAN, by reducing the protein mechanical stability via a direct interaction with the protein monomer, can favor the formation of oligomers that are known to aggregate into thicker amyloid fibrils. TMGA, by altering the protein monomer hydration shell, can favor the formation of proto-fibrils that are known to aggregate into thinner amyloid fibrils. In this latter case, each protein monomer can unfold completely, protected in a TMGA–water cage, before aggregating into proto-fibrils. The AFM investigation has also shown that, when the two ILs are mixed, the EAN mechanism of interaction prevails over that of TMGA. The huge variety of ILs and their well-established tailored-solvent character can offer, for instance, a new and vast landscape to tune protein amyloidogenesis, opening novel ways to use ILs in nanobio applications, including health and material sciences. For example, by inhibiting the formation of pathological protein aggregates, ILs can lead to the formulation of novel effective therapeutics, or by controlling amyloid fibrils’ mechanical properties, ILs can be exploited in advanced biomaterials.

**Table 1:** Average Values of Height and Surface Electric Potential of Lysozyme Amyloid Fibrils Incubated in Sole Water; Water Solutions of EAN, TMGA, and NaCl at a Molar Ratio of 3.5 ILs per Protein; and Water Solutions of EAN:TMGA at a Molar Ratio of 1:3.5^a^

	height (nm)	surface potential (V)	unfolding force (pN)
pure water/PBS	1.41 (0.11)	1.48 (0.01)	37 (7)
EAN	1.94 (0.05)	1.67 (0.01)	15 (7)
TMGA	1.12 (0.09)	0.67 (0.10)	42 (7)
NaCl	1.30 (0.08)	0.73 (0.05)	41 (7)
EAN:TMGA	1.77 (0.07)	1.57 (0.01)	n/a

a The average unfolding forces of lysozyme monomers in PBS and PBS solutions of EAN, TMGA, and NaCl at a concentration of 5% are also reported. The errors reported in parentheses represent one standard deviation.

## Supporting Information Available

Materials and methods, additional figures, and an additional table (PDF)
