# Supplementary material for: Controlling Amyloid Fibril Properties Via Ionic Liquids: The Representative Case of Ethylammonium Nitrate and Tetramethylguanidinium Acetate on the Amyloidogenesis of Lysozyme
Source: J Phys Chem Lett. 2022 Jul 28;13(30):7058–64. doi: 10.1021/acs.jpclett.2c01505 (PMC9358703; doi:10.1021/acs.jpclett.2c01505)
Supplement: Supplementary file 1 — jz2c01505_si_001.pdf [file jz2c01505_si_001.pdf]

Controlling Amyloid Fibril Properties Via Ionic Liquids: The Representative Case of Ethylammonium Nitrate and Tetramethylguanidinium Acetate on the Amyloidogenesis of Lysozyme

Visakh V.S. Pillai[1,2], Pallavi Kumari[1,2], Srikanth Kolagatla[1,2], Victoria Garcia Sakai[3], Svemir Rudić[3], Brian J. Rodriguez[1,2], Marina Rubini[4], Katarzyna M. Tych[5] and Antonio Benedetto[1,2,6,7*]

[1]School of Physics, University College Dublin, Dublin D04 N2E5, Ireland
[2]Conway Institute of Biomolecular and Biomedical Research, University College Dublin, Dublin D04 N2E5, Ireland
[3]ISIS Neutron and Muon Source, Rutherford Appleton Laboratory, Science & Technology Facilities Council, Didcot OX11 0QX, UK
[4]School of Chemistry, University College Dublin, Dublin D04 N2E5, Ireland
[5]Groningen Biomolecular Sciences and Biotechnology Institute, University of Groningen, 9747 AG Groningen, The Netherlands
[6]Department of Science, University of Roma Tre, 00146 Rome, Italy
[7]Laboratory for Neutron Scattering, Paul Scherrer Institute, 5232 Villigen, Switzerland
[*]Corresponding author: antonio.benedetto@ucd.ie ; antonio.benedetto@uniroma3.it ; antonio.benedetto@psi.ch

Supporting Information

Materials and Methods:

Materials:
Hen egg white Lysozyme (HEWL) with ≥95% purity, D_2O with ≥95% purity, NaCl, and PBS (tablets) were purchased from Sigma–Aldrich. For the optical tweezes experiments, one PBS tablet (Sigma–Aldrich catalogue number 79382) was dissolved in 200 mL milliQ water (18.2 MΩ·cm) to obtain a 137 mM NaCl, 2.7 mM KCl and 10 mM phosphate buffer solution (pH 7.4 at 25 °C). Ethyl ammonium nitrate (EAN) with >97% purity, and Tetramethyl guanidinium acetate (TMGA) with >95% purity were purchased from IOLITEC (Ionic Liquid Technologies). Plasmid T4 Lysozyme T21C K124C WT* was a gift from Brian Matthews (Addgene plasmid # 18243; http://n2t.net/addgene:18243; RRID:Addgene_18243). The DNA-coupling oligomers, biotin-modified primers, digoxigenin-modified primers and linker primers were ordered from IBA GmbH, Germany. The oxygen scavenging system components were glucose, glucose oxidase and glucose catalase. Glucose and glucose oxidase (Aspergillus niger) were purchased from Sigma-Aldrich. Glucose catalase was purchased from Serva (catalogue number 26910.01).

Amyloid fibrils preparation for Atomic Force Microscopy (AFM) and Open Loop Kelvin Probe Force Microscopy (OL-KPFM) experiments:
HEWL-water (ultra-pure with resistivity of 18.2 MΩ·cm) solutions (20 mg/mL) at a pH 2.0 (1.5 mL vial) in the presence and absence of EAN, TMGA and NaCl at concentrations of 1.0, 2.0, 3.5, and 5.0 ion pairs per protein were prepared. For the mixed IL experiments, addition of EAN was followed by addition of TMGA at EAN:TMGA molar ratios per protein of 1:x with x=1.0, 2.0, and 3.5. Amyloid fibrils were obtained after incubating the HEWL solutions at 65 ˚C, pH 2.0, and 350 rpm for 8 days (192 hours) in a thermomixer (Eppendorf). After 8 days of incubation, amyloid fibril solutions were centrifuged at 12,000 rpm for 20 min. Then 100 µL of solution was diluted 250 times and 150 µl was deposited onto a freshly cleaved mica support and kept for 5 min, followed by gentle rinsing with 1 mL of deionised water 3 times. Mica with amyloid fibrils was dried by storage overnight at room temperature followed by desiccation for 24 hours before use.

Atomic Force Microscopy (AFM) experimental set-up:

AFM imaging in air at ambient conditions was performed using a MFP-3D AFM (Asylum Research) operated in tapping (amplitude modulation) mode. Rectangular shaped Silicon probes with Al reflective coating (PPP-NCHR, Nanosensors) having a nominal tip radius of 7 nm were used. The force curve and thermal noise frequency methods were employed to calibrate the inverse optical lever sensitivity and spring constant of each cantilever (typically 42 ± 3 nN/nm).

Open Loop Kelvin Probe Force Microscopy (OL-KPFM) experimental set-up:

OL-KPFM measurements in air at ambient conditions were performed using an AFM (Asylum Research, MFP-3D) coupled with a lock-in amplifier (Zurich Instruments, HF2LI). Pt/Ir coated (PPP-EFM, Nanosensors) Silicon AFM probes with a nominal mechanical resonance frequency and spring constant of 75 kHz and 2.8 ± 0.46 nN/nm, respectively, were used. OL-KPFM is an open loop KPFM technique where DC bias is not applied. The first harmonic and second harmonic responses are collected and used to calculate the local Contact Potential Difference (CPD), also referred as "surface electric potential". CPD can be defined as the potential difference between the conducting AFM tip coating and the sample under investigation. For instance, higher CPD values correspond to higher electrostatic potential difference between the tip and the sample, hence to larger forces on the tip. For the OL-KPFM measurements, an electrical excitation frequency of 10 kHz at a time constant of 100 μs with an amplitude of 3 V was used. Similar to previous studies, the X gain value was kept equal to 1 for all measurements.[1]

Atomic Force Microscopy (AFM) data acquisition and analysis protocols:

For each investigated condition, two samples were prepared and 5×5 μm^2 AFM images were acquired at 3 different locations (at the corners) for each sample. Each AFM image was rendered and postprocessed using Asylum Research analysis software. Amyloid fibrils' heights were extracted from line profiles: 100 points were taken from each image and total of 600 points were considered for each investigated condition. The height distributions extracted from the 6 images acquired for the same condition were found to be overlapping, showing the reproducibility of the results. They have been combined together to compute the average height of the amyloid fibril for each condition, which followed a Gaussian distribution profile.

Open Loop Kelvin Probe Force Microscopy (OL-KPFM) data acquisition and analysis protocols:

For each investigated condition, two samples were prepared and OL-KPFM experiments were performed at 3 different locations (at the corners) for each sample. 5×5 μm^2 images were acquired for each sample. Along with the surface electric potential images, height images were obtained which were rendered and postprocessed using Asylum Research analysis software. Height images were masked and used to compute the surface electric potential distributions of the amyloid fibrils only excluding the substrate. The surface electric potential distributions extracted from the 6 images acquired for the same condition were found to be overlapping, showing the reproducibility of the results. They have been combined together to compute the average surface electric potential of the amyloid fibril at that condition. The surface electric potential has been also used to calculate the "work function" of the samples, which corresponds to the minimum thermodynamic work needed to remove an electron from the sample surface. A higher value of the work function means that it would take more energy to get an electron out of the sample surface. The work function of the sample was calculated by summing up the measured surface electric potential, multiplied by the electron charge, with the work function of the tip. The work function of the tip was measured to be 5.3 eV. This value has been obtained by measuring a calibration grid (Al, Si and Au metals) and using the theoretical work function of Si (i.e., 4.85 eV).[2]

Protein expression and purification for Optical Tweezers experiments:

The T4 Lysozyme variant bearing mutations T21C/C54T/K124C was produced using ER2566 *E. coli* cells transformed with plasmid T4 Lysozyme T21C/K124C WT*. The plasmid was a gift from Brian Matthews (Addgene plasmid #18243; http://n2t.net/addgene:18243; RRID:Addgene_18243). Protein production and purification was based on the protocols described elsewhere.[3] A single colony was used to inoculate 50 mL of LB broth supplemented with 100 µg/mL ampicillin. The cell culture was grown overnight at 37 °C and was used to inoculate (1:100) 2 L of fresh LB medium with 100 µg/mL ampicillin. Cells were shaken at 37 °C until an OD_{600} = 1.0 was reached. Protein expression was induced by addition of IPTG (final concentration 0.5 mM) and temperature was set to 30 °C. After 2.5 hours, cells were harvested by centrifugation at 4,500 rpm and the supernatant was discarded. The cell pellet was resuspended in 50 mL lysis buffer (0.1 M sodium phosphate buffer, pH 6.6, 0.2 M NaCl, 10 mM $MgCl_2$, 1 mM $CaCl_2$ and 5 mM EDTA). Cells were disrupted by sonication and the lysate was centrifuged for 90 min at 10,000 rpm and 4 °C to remove cell debris. Supernatants were dialyzed overnight against 50 mM Tris-HCl, pH 7.25 containing 1 mM EDTA. After centrifugation of the dialysate to remove aggregates, the solution containing the protein of interest was loaded onto a CM-Sepharose column, previously equilibrated with 50 mM Tris-HCl, pH 7.25 containing 1 mM EDTA. Lysozyme was eluted by applying a linear gradient of 10-500 mM NaCl in the same buffer. Fractions containing lysozyme were pooled, dialyzed against 50 mM sodium phosphate buffer, pH 6.7 and stored in this buffer at 4 °C.

Optical Tweezers experiments:

The two free cysteines in the T21C/C54T/K124C lysozyme were functionalised using maleimide-single-stranded DNA oligonucleotides. These single-stranded oligonucleotides were then hybridised with the single-stranded overhangs of 185-nm-long double-stranded DNA handles with either biotin or digoxygenin ends, resulting in a mixture of functionalised protein-DNA constructs of which 50% consist of one biotin DNA handle and one digoxigenin DNA handle attached on either side of the lysozyme molecule. These functionalised DNA handles will then bind to streptavidin- or antidigoxigenin-coated silica beads (1mm diameter, Spherotech, USA). The protein-DNA construct is incubated with a dilute solution of antidigoxigenin-coated silica beads for 15 minutes before being combined with a dilute solution of streptavidin-coated beads, the oxygen scavenging system (10 U/mL glucose oxidase, 100 U/mL glucose catalase and 0.1 % glucose final concentrations) and the buffer solution for the measurement. One of each bead type is trapped in the focal spots of each of the two parallel laser beams. The beads are then repeatedly brought together and separated at a constant velocity of 500 nm/s until a single protein-DNA construct tether is formed between them. Once the presence of a single tether is established, unfolding/refolding data is collected at a constant velocity of 500nm/s by repeatedly reducing and increasing the distance between the laser beams. The data were analysed by fitting unfolding/refolding force-extension traces using a worm-like-chain model to obtain the increases in contour length upon unfolding and the unfolding forces. Only traces with the expected change in contour length were analysed. The expected change in contour length is calculated as follows. The initial distance between the two cysteine residues based on the crystal structure is 3.1 nm. The number of amino acids between them is 103, so the expected increase in contour length is $(103 \cdot 0.35) - 3.1 = 36.05 - 3.1 = 32.95$ nm. The measured changes in contour length were in excellent agreement with this. For example, the pure PBS measurements gave an average increase in contour length of 32.1 ± 1.8 nm (mean ± SD), in excellent agreement with the expected length, for a total of 281 unfolding traces measured from three different molecules.

Elastic Neutron Scattering (ENS) experiments:

ENS data were collected on the time-of-flight neutron backscattering spectrometer IRIS[4] at the ISIS facility of the STFC Rutherford Appleton Laboratory, UK on the following samples: lysozyme/H_2O, lysozyme/D_2O, lysozyme/H_2O/EAN, lysozyme/D_2O/EAN, lysozyme/H_2O/TMGA, lysozyme/D_2O/TMGA, and vanadium (a good elastic incoherent scatterer used for data normalization). The hydration level was set to 0.4 grams of water per one gram of protein, which corresponds to the amount of water of the first protein hydration shell.[5] In the samples with ILs, the lysozyme:IL molecular ratio was 1:2. To minimize multiple scattering effects, the samples were filled into annular aluminium sample holders with an annular spacing of 0.2 mm to ensure a sample transmission of more than 90%. The IRIS spectrometer was used with the PG(002) analyser giving an energy resolution of 17 μeV (full width at half-maximum), and with a detector coverage spanning a wave vector transfer Q range of 0.5–1.8 $Å^{-1}$. Measurements were carried out at several temperatures in the range 10 K to 300 K. Standard data reduction was carried out using MANTID software.[6] For each sample and temperature, the ENS intensity was extracted from the measured quasi-elastic spectrum and summed in Q. The empty cell contribution was subtracted, and spectra were normalized to the vanadium standard.

Inelastic Neutron Spectroscopy (INS) experiments:

INS spectra in the 0–5,000 cm^{-1} range were recorded on the high-resolution indirect geometry inelastic spectrometer TOSCA at the ISIS facility of the STFC Rutherford Appleton Laboratory, UK, on water solutions of EAN and TMGA at 0.5 M, and on pure water for comparison.[7] The samples (ca. 2–3 g) were placed in a sealed flat thin-walled aluminium cans mounted perpendicular to the incident neutron beam using a standard TOSCA center stick. All the spectra were collected after the sample was cooled and stabilised at temperatures below 15 K with a closed-cycle refrigerator, and the spectra were recorded for a few hours. The Mantid software package was used to reduce and analyse the experimental data.[8]

Figures & Tables:

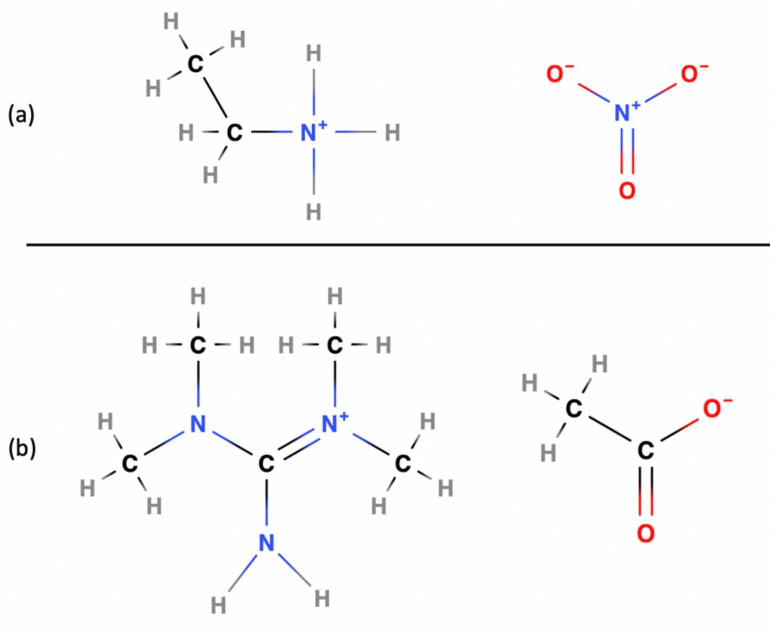

SI Fig. 1 – Schematic chemical structure representations of (a) Ethyl ammonium nitrate (EAN) and (b) Tetramethyl guanidinium acetate (TMGA).

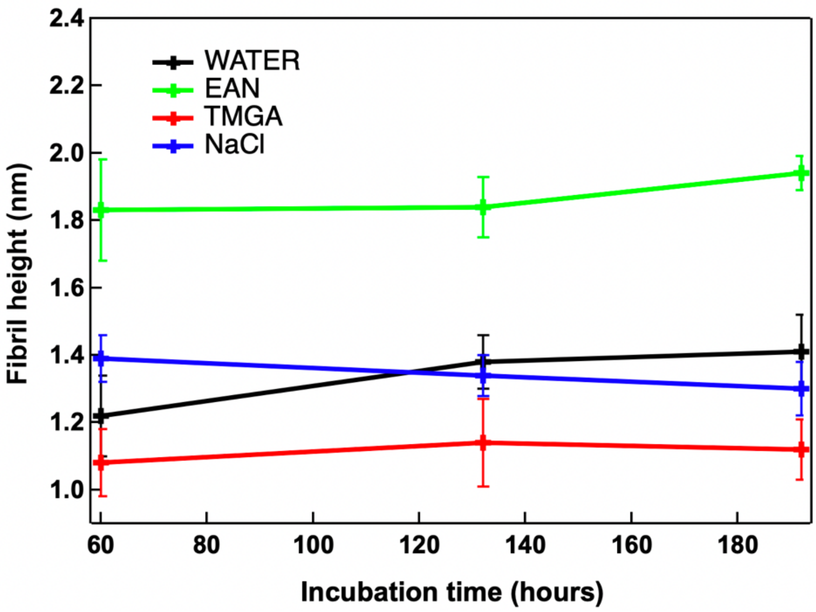

SI Fig. 2 – Average height values as a function of incubation time for lysozyme amyloid fibrils incubated in water solutions of EAN (green), TMGA (red) and NaCl (blue) at 3.5 ion pairs per protein along with its value upon incubation in sole water (black line). Error bars are one standard deviation. Continuous lines are guides for the eye.

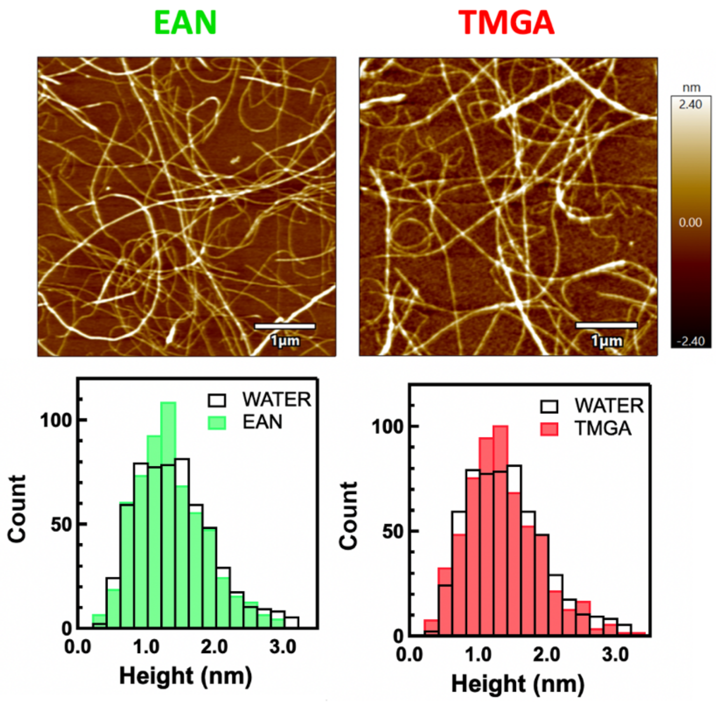

SI Fig. 3 – Representative height's AFM images and associated histogram distributions of lysozyme amyloid fibrils incubated first in sole water for 8 days (at 65 ˚C and pH 2.0) and then in water solutions of EAN (green) and TMGA (red) at a concentration of 3.5 ILs per protein for 8 additional days at room temperature. The histogram distributions are not limited to the data of the representative figures but have been obtained using the all sets of AFM data (see *SI Materials and Methods* section for more details). This additional set of AFM data shows that the addition of the two ILs to sole water-incubated mature amyloid fibrils does not have any effect on amyloid fibrils' height.

	Work function (eV)
**Pure water**	3.82 (0.05)
**EAN**	3.63 (0.05)
**TMGA**	4.63 (0.5)
**NaCl**	4.57 (0.2)
**EAN:TMGA**	3.73 (0.05)

SI Tab. 1 – Average values of work function of lysozyme amyloid fibrils incubated in sole water, water solutions of EAN, TMGA and NaCl at a concentration of 3.5 ion pairs per protein, and water solutions of EAN:TMGA at 1:3.5 molar ratio. The errors reported in brackets represent one standard deviation.

Bibliography

(1) Collins, L.; Kilpatrick, J. I.; Weber, S. A. L.; Tselev, A.; Vlassiouk, I. V.; Ivanov, I. N.; Jesse, S.; Kalinin, S. V.; Rodriguez, B. J. Open Loop Kelvin Probe Force Microscopy with Single and Multi-Frequency Excitation. *Nanotechnology* **2013**, *24* (47), 475702.

(2) Melitz, W.; Shen, J.; Kummel, A. C.; Lee, S. Kelvin Probe Force Microscopy and Its Application. *Surf. Sci. Rep.* **2011**, *66* (1), 1–27.

(3) Poteete, A. R.; Sun, D. P.; Nicholson, H.; Matthews, B. W. Second-Site Revertants of an Inactive T4 Lysozyme Mutant Restore Activity by Restructuring the Active Site Cleft. *Biochemistry* **1991**, *30* (5), 1425–1432.

(4) Carlile, C. J.; Adams, M. A. The Design of the IRIS Inelastic Neutron Spectrometer and Improvements to Its Analysers. *Phys. B: Condens. Matter* **1992**, *182* (4), 431–440.

(5) Benedetto, A. Low-Temperature Decoupling of Water and Protein Dynamics Measured by Neutron Scattering. *J. Phys. Chem. Lett.* **2017**, *8* (19), 4883–4886.

(6) Taylor, J.; Arnold, O.; Bilheaux, J.; Buts, A.; Campbell, S. I. Mantid, A High Performance Framework for Reduction and Analysis of Neutron Scattering Data. *Bull. Am. Phys. Soc.* **2012**, *57*, W26.

(7) Pinna, R. S.; Rudić, S.; Parker, S. F.; Armstrong, J.; Zanetti, M. The Neutron Guide Upgrade of the TOSCA Spectrometer. *Nucl. Instrum.* **2018**, *896*, 68–74.

(8) Arnold, O.; Bilheux, J. C.; Borreguero, J. M.; Buts, A.; Campbell, S. I. Mantid—Data Analysis and Visualization Package for Neutron Scattering and µ SR Experiments. *Nucl. Instrum.* **2014**, *764*, 156–166.
